# Role of cellulose and its derivatives for 3D printing of bone tissue scaffolds

**DOI:** 10.3389/fbioe.2026.1814617

**Published:** 2026-07-06

**Authors:** Duygu Ege, Ali Reza Kamali, Stuart Goodman

**Affiliations:** 1 Institute of Biomedical Engineering Boğaziçi University Istanbul, Istanbul, Türkiye; 2 School of Metallurgy, Northeastern University, Shenyang, China; 3 Department of Orthopeadic Surgery, Stanford Medicine, Stanford, CA, United States

**Keywords:** bacterial cellulose, carboxymethyl cellulose, cellulose nanocrystal, cellulose nanofiber, methyl cellulose

## Abstract

3D printing has become central to bone tissue engineering, yet many printable polymers lack the mechanical strength, hydrophilicity, and bioactivity needed for effective bone regeneration. Cellulose and its derivatives have emerged as versatile additives that address these limitations across multiple scaffold types. This review highlights recent advances in 3D printed scaffolds incorporating cellulose nanocrystals (CNC), microcrystals (MCC), nanofibrils (CNF), bacterial cellulose (BC), and chemically modified cellulose derivatives including carboxymethyl cellulose (CMC), methyl cellulose (MC), diethylaminoethyl-modified cellulose (DEAE) and cellulose acetate (CA). In hydrogels, CNF and BC provide shear-thinning behavior and mechanical strength, while in polymer–ceramic hybrids, CNC improves stiffness. CA, owing to its thermoplastic processability and tunable hydrophobicity, enables high-resolution extrusion and melt-based printing while serving as a mechanically stable backbone that can be further biofunctionalized through surface modification. CMC, MC and DEAE cellulose are key printing enablers when blended with polymers such as gelatin or HAp. This review also shows that further modification of cellulose seems to enhance its potential for future clinical translation. Together, these findings demonstrate that cellulose is a key design element that transforms mechanically weak or biologically inert inks into robust, osteoconductive scaffolds. The review concludes with emerging trends and remaining opportunities for cellulose-based bioinks in bone regeneration.

## 1 Introduction

Bone defects caused by trauma, tumor resection, infection, and congenital abnormalities remain a major clinical challenge, and current gold-standard treatments such as autografts and allografts are limited by donor site morbidity, finite availability, and risks of immune rejection or disease transmission (V Murphy and Atala, 2014; Ege et al., 2014). In this context, bone tissue engineering aims to develop 3D scaffolds that not only provide mechanical support but also guide cell adhesion, proliferation, differentiation, and matrix mineralization. In recent years, 3D printing technologies have become central to this effort, enabling the fabrication of patient-specific constructs with precisely controlled pore architecture, spatially graded compositions, and reproducible mechanical properties (Xu et al., 2021). However, many of the polymers that are most compatible with 3D printing (e.g., PCL, PLA, medical-grade resins) are hydrophobic and bioinert and therefore require compositional or surface modification to achieve satisfactory bioactivity and osteogenic performance (Woodruff and Hutmacher, 2010).

**FIGURE 1:**
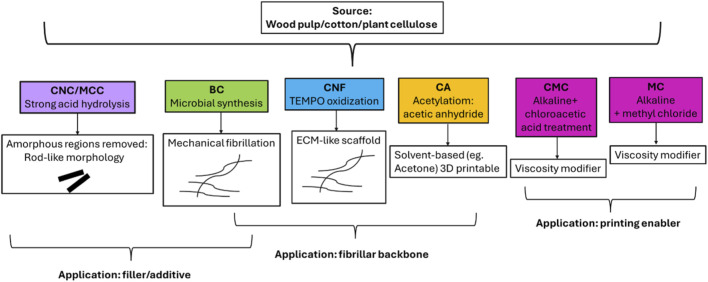
Different cellulose types including CNC/MCC (Habibi et al., 2010), CNF (Isogai et al., 2011), BC, CMC (Heinze and Koschella, 2005), MC (Nasatto et al., 2015) and CA (Edgar et al., 2001), their processing conditions and general properties.

Within this context, cellulose and its derivatives have emerged as highly versatile components for the design of 3D printed scaffolds (Khalid et al., 2023; Jan et al., 2023). Cellulose is generally extracted from wood pulp, cotton or plants (Moon et al., 2011). Owing to its abundance, cytocompatibility, chemical modifiability, and rich hierarchical structure, cellulose can be introduced in different forms from nanocrystals (CNC), microcrystalline particles (MCC) to nanofibrils (CNF), chemically modified soluble derivatives (carboxymethyl cellulose (CMC) and methyl cellulose (MC)), bacterial cellulose (BC) and cellulose acetate (CA) to perform multiple roles within scaffold formulations (Moon et al., 2011; Klemm et al., 2011). Figure 1 shows different cellulose types and their processing conditions.

As shown in Figure 1, CNC and MCC are produced by acid hydrolysis of wood pulp, cotton or plant cellulose (Habibi et al., 2010). A growing body of work has shown that CNC and MCC can act as stiff, rod-like reinforcements that increase the stiffness and mechanical strength of synthetic polymer matrices, improve hydrophilicity and protein adsorption, and promote HAp nucleation in polylactic acid (PLA) and polycaprolactone (PCL)-based composites and hybrid systems (Habibi et al., 2010). In both synthetic (PLA, PCL, resins) and natural (gelatin, alginate, collagen) matrices, low-to-moderate CNC or MCC loadings typically enhance printability, structural stability, and osteogenic cell responses, while surface coatings of CNCs offer an additional route to tune nanotopography and chemical functionality without altering bulk mechanics (Khalid et al., 2023; Jan et al., 2023).

**FIGURE 2:**
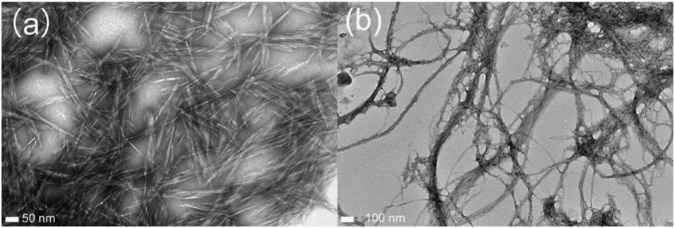
High-resolution TEM images of **(a)** CNCs and **(b)** CNFs (Xu et al., 2013). Reproduced with permission from Ref. (Xu et al., 2013).

Cellulose nanofibrils (CNF) are produced by mechanical fibrillation of purified cellulose fibers, often assisted by chemical or enzymatic pre-treatments, yielding long, flexible nanofibrils containing both crystalline and amorphous domains (Isogai et al., 2011). Cellulose microfibers and nanofibrils (CNFs) have been exploited primarily as network-forming, rheology-controlling elements in hydrogels and mineralized composites (De France et al., 2017). TEMPO-oxidized CNFs provide highly entangled nanofibrous backbones that confer shear-thinning behavior, viscosity recovery, and shape fidelity to alginate, gelatin, peptide, and double-network hydrogels, thereby enabling stable 3D printing (Raveendran et al., 2025). These CNF-reinforced systems generally maintain robust architecture after crosslinking, show markedly improved tensile or compressive properties, and, when combined with bioactive phases such as HAp or calcium-deficient HAp, support biomimetic mineralization and osteogenic differentiation (Gorgieva et al., 2017). Figure 2 shows high-resolution TEM images of pristine (a) CNCs and (b) CNFs (Xu et al., 2013).

Additionally, soluble, chemically modified cellulose such as CMC, MC, and CA have been used to tailor crosslinking chemistry, gelation behavior, injectability, and drug delivery. CMC has served as both a soluble matrix and a rheology modifier where it enables shear-thinning direct-ink writing, dual-porous architectures, and sustained release of bioactive molecules while maintaining high cell viability (Patel et al., 2021; N’Gatta et al., 2022; Leonovich et al., 2023; Alemán‐Domínguez et al., 2019; N’Gatta et al., 2025; Averianov et al., 2022; Giubilini et al., 2023).

**FIGURE 3:**
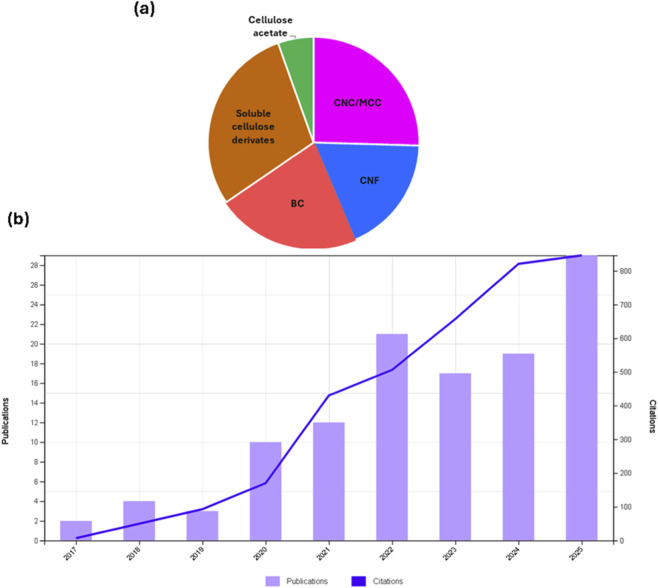
**(a)** Percentage of different types of cellulose for 3D printing bone tissue engineering scaffolds (drawn in Microsoft Excel from extracted data in literature) **(b)** Number of published papers yearly in cellulose-based nanomaterials for 3D printing bone tissue engineering scaffolds. (extracted from Web of Science in 2025 using the search string: TS = (cellulose AND “3D printing and 3D print”).

Finally, BC composites bring yet another dimension to cellulose-based design. BC membranes and micronized BC reinforcements have improved the mechanical performance, surface hydrophilicity, and drug-loading capacity of biodegradable polymers, and have demonstrated superior bone formation and infection control *in vivo* defect models (Saska et al., 2011). Together, these advances indicate that cellulose is no longer a passive filler but a central design element that can simultaneously address mechanical, rheological, biological, and even immunomodulatory requirements in 3D printed bone scaffolds. Figure 3a shows the literature found for types of cellulose used for 3D printing bone tissue engineering scaffolds and Figure 3b illustrates the number of published papers yearly in cellulose-based nanomaterials for 3D printing bone tissue engineering scaffolds. Ongoing research is active for all cellulose types for production of bone scaffolds by 3D printing methods for all of the mentioned types of cellulose.

This review synthesizes the rapidly expanding literature on cellulose-based 3D printed scaffolds for bone tissue engineering. We first discuss CNCs and MCCs in polymer and hybrid composites, then examine cellulose microfibers and CNFs in hydrogel and mineralized systems, followed by CMC and other soluble derivatives, CA and related high-viscosity polymers, and, finally, BC constructs. Across these classes, we highlight common structure–property–function relationships, identify optimal cellulose forms and loadings for enhancing printability and osteogenic performance, and outline remaining challenges and future directions for cellulose-containing 3D printed scaffolds in bone regeneration.

## 2 3D printed bone tissue engineering exploiting various types of cellulose

We performed a literature search from 2000 through 2025 with the search engines Web of Science, Scopus, and Google Scholar. The search terms were as follows: cellulose, 3D printing, bone tissue engineering, cellulose nanocrystals, microcrystalline cellulose, cellulose nanofibers, carboxymethyl cellulose, methyl cellulose, cellulose acetate and bacterial cellulose. In this study, work in this field is demonstrated and categorized which is discussed in sections including CNC, CNF, BC, modified soluble cellulose types (CMC, MC and DEAE) and CA.

### 2.1 CNC and MCC in 3D printing for bone tissue engineering

For 3D printing bone tissue engineering scaffolds, CNCs are widely used as multifunctional reinforcements across polymeric systems, consistently improving rheology and print fidelity (Dobaj Štiglic et al., 2023; Mahmoudi et al., 2025; Dutta et al., 2021; Patel et al., 2021), mechanical stiffness and structural stability (Mahmoudi et al., 2025; N’Gatta et al., 2022; Leonovich et al., 2023; Alemán‐Domínguez et al., 2019), and surface hydrophilicity (N’Gatta et al., 2025; Averianov et al., 2022; Giubilini et al., 2023). CNCs also enhance apatite formation and mineral deposition when 3D printed with PLA, PCL, and hydrogel systems (Mahmoudi et al., 2025; Dutta et al., 2021; Patel et al., 2021; N’Gatta et al., 2022; N’Gatta et al., 2025; Janmohammadi et al., 2025). These recurring effects explain why CNCs are widely adopted for improving both printability and biological performance in a variety of scaffold architectures.

**FIGURE 4:**
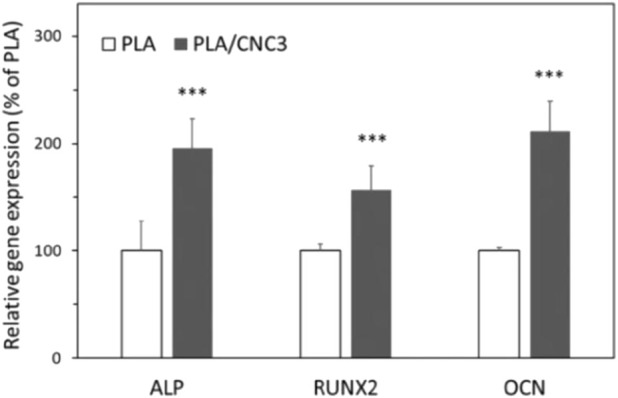
Expression of ALP, RUNX2 and OCN osteogenic differentiation marker genes on hFOB cells grown for 2 days in presence of PLA and PLA/CNC3 eluates. Statistical analysis has been performed using the Mann–Whitney test between PLA and PLA/CNC3 (***, *p* < 0.001) (N’Gatta et al., 2022). Reproduced from Ref. (N’Gatta et al., 2022), Scientific Reports (2022), CC BY 4.0.

As shown in Figure 4, polyesters were often loaded with CNCs and 3D printed to improve their mechanical behavior and cellular response (N’Gatta et al., 2022; Leonovich et al., 2023). Table 1 also shows details of the prepared scaffolds (including composition and 3D printing method and temperature) and CNC’s effects on the 3D printed bone tissue regeneration scaffolds. PLA is one of the common exploited polymers for this purpose. N’Gatta *et al.* (N’Gatta et al., 2022) developed 3D printed composite scaffolds using PLA reinforced with upto 5 wt% CNCs (PLA/CNC3) to enhance mechanical, structural properties and bioactivity for bone tissue engineering. The addition of 3 wt% CNC increased stiffness by ∼30% and improved hydrophilicity, producing scaffolds with ∼400 µm interconnected pores and superior surface wettability. However, 5 wt% of CNC reduced the tensile strength due to agglomeration. CNC incorporation promoted HAp nucleation during SBF exposure, indicating enhanced bioactivity and mineralization potential. *In vitro* studies using hFOB osteoblasts confirmed that PLA/CNC scaffolds were cytocompatible, supporting cell adhesion, spreading, and proliferation.

**TABLE 1:** 3D printed CNC or MCC incorporated scaffolds for bone tissue engineering applications.

Second main component	Composition (printing method, temperature)	Effect of CNC
PLA	3 wt% CNC/PLA (FDM, 190 °C–210 °C)	CNC increased stiffness and improved hydrophilicity. It also promoted HAp nucleation (N’Gatta et al., 2022)
	Methacrylated CNC/PLA (extrusion-based)	CNC increased stiffness and printing stability. CNC facilitated MSC adhesion (Leonovich et al., 2023)
PCL	5 wt% CNC/AgNP PCL (FDM, 80 °C–100 °C)	CNC enhanced hydrophilicity and HAp nucleation (N’Gatta et al., 2025)
	5–20 wt% MCC/PCL (FDM, 80 °C–90 °C)	MCC improved mechanical properties, increased hydrophilicity, filament stability and promoted MC3T3-E1 cell adhesion (Alemán‐Domínguez et al., 2019)
	1–5 wt% CNC/poly (glutamic acid) (PGA)/PCL (FDM, 80 °C–100 °C)	CNC increased compressive modulus and strength. Enhanced hydrophilicity and apatite formation. Augmented compressive strength and modulus. 1–3 wt% CNC had highest cell adhesion and mineralization (Averianov et al., 2022)
	1–5 wt% CNC/BG/traganth gum/PCL (FDM, 80 °C–100 °C)	CNC boosted swelling, hydrophilicity, apatite formation. Enhanced compressive strength and modulus. 1–3 wt% CNC had highest cell adhesion and mineralization (Janmohammadi et al., 2025)
	0.5–2 wt% CNC coating on PCL (FDM, 80 °C–90 °C)	CNC increased surface roughness, osteoblast adhesion (Babi et al., 2021)
Collagen/Gelatin	1 wt% CNC/collagen (DIW, 20 °C–25 °C)	CNC improved viscosity, filament stability, shape fidelity and enhanced stiffness (Dobaj Štiglic et al., 2023)
	1–5 wt% CNC/gelatin/HAp (DIW, 30 °C–40 °C)	Improved viscosity, filament fidelity, stability enhanced stiffness and bioactivity (Mahmoudi et al., 2025)
Alginate	1–5 wt% CNC/alginate/gelatin (DIW, 25 °C–37 °C)	CNC enhanced printability, stability, filament stability (Dutta et al., 2021)
	2 wt% CNC/alginate/gelatin (DIW, 25 °C–37 °C)	CNC improved printability, stability and biological performance, filament stability, swelling capacity, improved hBMSCs cell viability and spreading. It also upregulated osteogenic markers (Patel et al., 2021)
Glycerophosphate	0.5, 1 w/v% CNC/glycerophosphate/hydroxyethyl cellulose (DIW, 37 °C)	Improved shape fidelity, mechanical integrity, osteogenic differentiation enhanced (Maturavongsadit et al., 2021)
Silk fibroin	Cellulose/collagen, silk fibroin (DIW, 25 °C–37 °C)	ECM mimicry scaffold (Ramanathan et al., 2023)
	Cellulose nanoparticle/chitosan/silk fibroin (DIW, 25 °C–37 °C)	Enhanced rheological properties, swelling capacity, and induced immunomodulatory effect. New bone formation occurred (Patel et al., 2022)
PHBH	PHBH/cellulose (FDM, 150 °C–170 °C)	Promoted printability, mechanical strength, stiffness, structural stability, surface roughness, interlayer bonding (Giubilini et al., 2023)

Nonetheless, as shown in Figure 4, 3 wt% CNC incorporation significantly improved alkaline phosphatase (ALP), Runt-related transcription factor 2 (RUNX2) and osteocalcin (OCN) expression.

Researchers have incorporated CNCs into PCL scaffolds through several strategies including bulk, hybrid scaffolds and as coatings (Alemán‐Domínguez et al., 2019). MCC which is a hydrophilic and highly crystalline cellulose phase, was also used for preparing 3D printed scaffolds (Alemán‐Domínguez et al., 2019). Alemán‐Domínguez et al. (2019) produced scaffolds by melt-mixing PCL with 5–20 wt% MCC and fabricating the composites using extrusion-based 3D printing. The inclusion of MCC markedly improved mechanical stiffness and compressive strength, with the most pronounced reinforcement observed in the 10–15 wt% range where cellulose dispersed well without severe agglomeration. MCC also increased surface hydrophilicity, reducing water contact angles and promoting greater protein adsorption compared to neat PCL. During printing, the cellulose phase enhanced filament stability and dimensional accuracy, producing more defined struts and pore uniformity. Overall, cell culture studies with Presto Blue® assay showed that on days 1, 3 and 8, MCC loading improved MC3T3-E1 pre-osteoblastic cell adhesion, spreading confirming cellulose as the dominant contributor to improved cytocompatibility.

CNCs improve printability and structural fidelity in soft materials where mechanical reinforcement is otherwise limited. In collagen or gelatin, alginate/gelatin, chitosan-based systems CNC has been incorporated. In collagen and gelatin-based systems, CNCs increase viscosity, filament stability, and post-printing integrity, while supporting improved cell adhesion and proliferation in collagen-only systems (Dobaj Štiglic et al., 2023) and gelatin (Type A, gel strength of 300 Bloom)/HAp scaffolds (Mahmoudi et al., 2025). In alginate–gelatin (Type B, gel strength of 300 g bloom hydrogels), addition of one to five wt% CNC enhanced rheology, mechanical stability, and swelling characteristics while promoting mineral deposition and osteogenic marker expression (Dutta et al., 2021; Patel et al., 2021). In chitosan-based bioinks, CNCs enhance gelation kinetics, structural integrity, and ECM deposition (Maturavongsadit et al., 2021; Im et al., 2022). Across all natural biomaterials, CNCs function as essential reinforcing nanofillers that enable more stable, bioactive hydrogel scaffolds.

**FIGURE 5:**
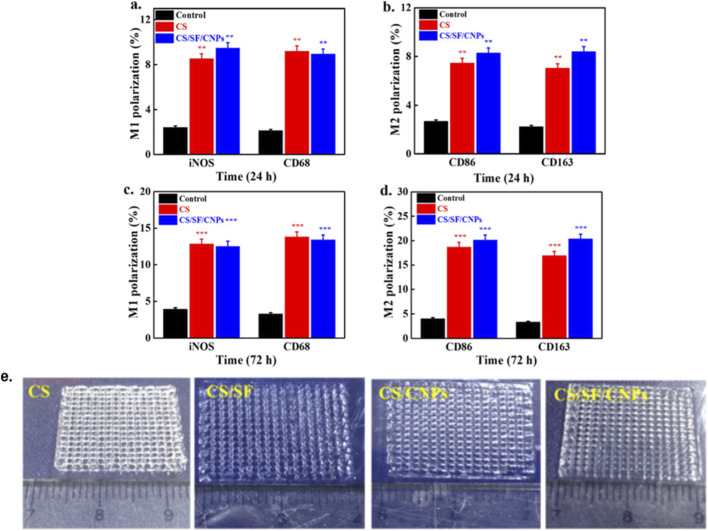
**(a–d)** Evaluation of the macrophage polarization potential of the printed scaffolds with Raw 264.7 cells by the FACS technique after 1 and 3 days of treatment and **(e)** 3D printed constructs (Patel et al., 2022). Reproduced with permission from Ref. (Patel et al., 2022).

Patel et al. (2022) fabricated 3D printable chitosan/silk fibroin scaffolds reinforced with CNC showed that incorporation of CNC markedly improved the rheological behavior, recovery strength, swelling capacity and degradation of the hydrogels compared with pure polymer scaffolds. Interestingly, CNC/chitosan/silk fibroin scaffolds induced a pronounced immunomodulatory effect, promoting a significant shift in macrophage polarization from pro-inflammatory M1 to pro-healing M2 after 3 days, indicating that the cellulose-containing composite can actively tune the immune microenvironment. Conditioned media from macrophages cultured on the CNC incorporated scaffolds enhanced the osteogenic differentiation of hBMSCs, with upregulated osteogenic markers and increased mineralization. In a rat calvarial defect model, as shown in Figure 5, CNC incorporated scaffolds led to substantially greater new bone formation and defect bridging than controls, confirming that the immunomodulation (M2 polarization) translated into improved *in vivo* bone regeneration. Overall, the study highlights CNC as key contributors to mechanical reinforcement and osteo-immunomodulatory behavior, making CNC incorporated scaffolds promising candidates for macrophage-guided bone repair.

Surface modification of CNCs has emerged as a crucial strategy to address dispersion limitations, improve polymer compatibility, and introduce new chemical functionalities. Methacrylated CNCs were integrated into methacrylated PLA networks, enabling CNCs to participate directly in covalent crosslinking during photopolymerization (Leonovich et al., 2023). This dual role including mechanical reinforcement and network integration produced scaffolds with improved structural organization and printing stability. Up to day 3, cell studies with NIH/3T3 fibroblasts and human MSCs demonstrated excellent cytocompatibility, with MSCs exhibiting superior adhesion and spreading on the 3D printed CNC-containing scaffolds. Overall, the work highlights that CNC enhances mechanical performance, supports stable printing, and promotes favorable cell–scaffold interactions in PLA-based systems.

In another study, poly (glutamic acid) (PGA) grafting significantly improved CNC dispersion in hydrophobic PCL, resulting in a higher compressive modulus, improved hydrophilicity, and better protein adsorption (Averianov et al., 2022). The study evaluated *in vitro* performance using a standard critical-sized calvarial defect model using Sprague–Dawley rats, with scaffolds implanted into bilateral 8-mm defects in the parietal bone. Histological analysis showed that PCL/PGA-CNC scaffolds supported significantly greater new bone formation than pristine PCL, and the rMSC-seeded PCL/PGA-CNC constructs yielded the highest bone tissue area at both 30 and 90 days, confirming the enhanced osteogenic capacity of the CNC-reinforced system in a non-weight-bearing cranial bone environment. Overall, PGA-CNC addition accelerated degradation, improved cell adhesion, and enhanced early osteogenic activity confirming the cellulose phase as the major contributor to mechanical, interfacial, and biological improvements. This study is one of the most compelling demonstrations of CNC-driven enhancement in bone regeneration.

### 2.2 BC in 3D printing bone tissue engineering scaffolds

BC has emerged as one of the most versatile cellulose-based components in 3D printed bone scaffolds, distinguished by its high crystallinity, exceptional water-holding capacity, nanofibrillar morphology, and inherent cytocompatibility. Across diverse fabrication approaches from polymer–BC composites (Cakmak et al., 2020), BC-coated thermoplastic scaffolds (Jiang et al., 2025), and PLA-on-BC hybrid structures (Wu et al., 2022) to BC-reinforced hydrogels (Wang et al., 2021) and chemically modified BC bioinks (Wang et al., 2024). BC consistently serves as a multifunctional structural and biological enhancer. Its ultrafine nanofibrillar network provides ECM-like topography, high surface area, and robust hydration, enabling strong improvements in osteoblast adhesion, proliferation, and osteogenic differentiation (Cakmak et al., 2020; Aki et al., 2020). It usually serves as a filler/additive similar with CNC and MCC. At the same time, BC enhances printability, mechanical stability, and hydrogel integrity, whether incorporated as dispersed nanofibers as a continuous membrane, or as the primary hydrogel backbone (Jiang et al., 2025; Aki et al., 2020; Iglesias-Mejuto et al., 2025). Table 2 shows 3D printed BC-based scaffolds.

**TABLE 2:** 3D printed BC incorporated scaffolds for bone tissue engineering applications.

Main additional component	Composition	Benefits of bacterial cellulose
PCL	0.25 HAp/BC/PCL/gelatin (FDM, 80 °C–100 °C)	BC improved hydrophilicity, improved cell interactions (Cakmak et al., 2020)
	PCL coated with BC membrane/psoralen (FDM, 80 °C–90 °C)	BC improved hydrophilicity, cell interactions and osteogenic RUNx2 and VEGF expression increased (Jiang et al., 2025)
PLA	BC/PLA (FDM, 190 °C–210 °C)	High cytocompatibility (Wu et al., 2022)
Gelatin	BC/gelatin (DIW, 25 °C–37 °C)	BC provided highly entangled network. Provides shear thinning properties and viscosity, provided shape fidelity stability (Wang et al., 2021)
	Maleic acid modified BC/gelatin (DIW, 25 °C–37 °C)	Maleic acid upregulated Alp, Runx2, Col1 [39]
PVA	0.25 wt% h-BN/0.5 wt% BC/PVA (DIW, 25 °C–40 °C)	BC nanofibrous, hydrophilic reinforcement improved ductility. Osteoblast viability increased (Aki et al., 2020)
MC	Polyurea crosslinked BC nanofiber/MC (DIW, 25 °C)	BC stiffened the network, reduced shrinkage (Iglesias-Mejuto et al., 2025)
HaP	BC/HAp (Ink-jet printing, ambient temperature)	BC acted as structural backbone, enabled printability (Liu et al., 2024)
Probiotic embedded	Probiotic embedded BC scaffold (DIW, 25 °C)	BC acts as nanofibrillar backbone (Sabio et al., 2023)
PHB	0.25–2 wt% micronized BC/PHB (FDM, 160 °C–180 °C)	BC improved mechanical properties (elastic modulus), BC enhanced hydrophilicity and micro-roughness enhanced L929 fibroblast adhesion (Celestino et al., 2023)

Interestingly, in some studies BC is used as an additive and in other studies it is used as the backbone (structural matrix) of the prepared scaffolds. In several studies BC is used as a filler where it is used combined with polymers such as PCL. For instance, Cakmak et al. (Cakmak et al., 2020) fabricated 3D printed composite scaffolds based on PCL/gelatin (gel strength ∼300 g Bloom, Type A) and incorporated 1 wt% BC nanocrystals and 0.25 wt% HAp to improve suitability for bone tissue engineering. The printing process yielded scaffolds with pore sizes around 300 μm, within the range considered favorable for vascularized bone ingrowth. The addition of BC and HAp reduced the tensile strength compared to PCL/GEL alone. The reduction in tensile strength upon incorporation of BC and HAp has been attributed to different mechanisms. Specifically, BC was reported to induce weak physical interactions between polymer chains under loading, while the presence of HAp led to imperfect interlayer bonding along the z-axis during the printing process, resulting in reduced tensile performance. BC/PCL/GEL and particularly 0.25 wt% HaP/BC/PCL/GEL scaffolds significantly improved MC3T3-E1 pre-osteoblast cell viability, adhesion, and spreading, as evidenced by MTT assays and SEM, where MC3T3-E1 pre-osteoblast cells exhibited well-spread morphologies with pseudopodia-like extensions across the scaffold surface on day 1, 4 and 7 of the cell culture studies. The presence of BC contributed a hydrophilic, ECM-like nanofibrillar phase that enhanced cell–scaffold interactions, while HAp further boosted osteoconductivity and metabolic activity. Overall, the study showed that integrating BC into a PCL/GEL matrix transformed a purely synthetic scaffold into a more cell-responsive, bioactive composite, with the 0.25%HaP/BC/PCL/GEL formulation offering the best balance between structural properties and osteoblast response for bone tissue engineering.

Aki et al. (2020) 3D printed composite scaffolds based on polyvinyl alcohol (PVA, MW = 89,000–98,000), hexagonal boron nitride (hBN) and BC filler for bone tissue engineering. The optimal formulation contained 12 wt% PVA, 0.25 wt% hBN, and 0.5 wt% BC in the printing blend. BC was homogeneously dispersed in the PVA/hBN matrix and acted primarily as a nanofibrous, hydrophilic reinforcement. Its incorporation changed the blend morphology, increased swelling capacity, and significantly improved ductility, although tensile strength decreased slightly compared with BC-free PVA/hBN scaffolds. From a biological standpoint, human osteoblast cell viability and attachment were highest on the BC-doped scaffolds, and SEM images showed well-spread osteoblasts tightly adhered to the BC-containing surfaces. Overall, the work demonstrated that adding 0.5 wt% of BC to a PVA/hBN matrix transformed it into a more compliant, hydrated, and osteoblast-friendly 3D printed scaffold with strong potential for bone tissue engineering applications.

Celestino et al. (2023) developed 3D printable filaments by melt-compounding polyhydroxybutyrate (PHB) with 0.25–2.0 wt% micronized BC, producing biodegradable scaffolds suitable for general tissue-engineering applications. The addition of BC significantly improved the mechanical properties of PHB, increasing elastic modulus and tensile performance while maintaining good printability. BC also enhanced surface hydrophilicity and micro-roughness, resulting in improved protein adsorption and markedly better fibroblast adhesion compared to neat PHB. On day 1 and 3 of the cell culture studies, the composite filaments were non-cytotoxic and supported L929 fibroblast viability, confirming their suitability for soft-tissue or connective-tissue scaffold applications. Overall, the BC phase acts as a multifunctional reinforcement that improves mechanical strength, wettability, and biological performance in PHB-based 3D printed constructs.

BC was also used as a coating in literature. Jiang et al. (2025) designed scaffolds by 3D printing PCL and then forming a BC membrane loaded with the natural antimicrobial compound psoralen over the scaffold, achieving a multilayer composite structure. The BC phase served as a high-water-content, highly porous, and cytocompatible matrix that enabled efficient psoralen loading and sustained release. Incorporation of BC markedly improved surface hydrophilicity and cellular interactions, supporting better rat BMSC adhesion, proliferation, and osteogenic differentiation compared to pure PCL scaffolds. The BC/psoralen layer also provided strong antibacterial activity, particularly against *S. aureus*, addressing infection risks inherent to open bone defects. Micro-CT and histology studies were conducted in a rat critical-sized calvarial defect model. Immunohistochemistry demonstrated significantly higher RUNX2, COL2, OCN and VEGF expression in BC/Pso/PCL group compared to pure PCL. Overall, the BC/Pso/PCL composite achieved superior bone formation *in vivo*, demonstrating that the cellulose phase is essential for drug delivery, improved biointerface properties, and enhanced regenerative performance.

In many studies, BC was also used as the structural backbone. In such cases, BC is usually combined with a soft polymer such as gelatin. Wang et al. (2021) formulated a 3D printable hydrogel bioink by combining gelatin with BC to obtain a mechanically robust, cell-adhesive gel suitable for bone regeneration. In this system, BC provides the nanofibrillar backbone where its highly entangled network supplies shear-thinning behavior and viscosity sufficient for extrusion printing, while helping the printed constructs maintain shape fidelity and structural stability, which pure gelatin cannot achieve on its own. Gelatin contributes cell-recognition motifs and degradability, whereas BC reinforces the hydrogel, improving mechanical strength and resistance to collapse during and after printing. *In vivo*, the printed BC/gelatin constructs implanted into bone defects supported substantial new bone formation, with enhanced mineralization and tissue ingrowth compared to controls, indicating that the BC-reinforced gel provided a favorable microenvironment for osteogenesis. Overall, the study showed that BC is the key structural component that converts a soft gelatin gel into a printable, load-sharing, and osteoconductive scaffold capable of promoting *in vivo* bone regeneration.

In another study, the authors constructed an osteo-inductive bio-ink by chemically modifying BC with maleic acid (MA) and then hybridizing the modified BC (MA-BC) with gelatin. BC was treated in 60 w/v% MA solution at different solid–liquid ratios (BC:MA solution from 1:5 to 1:50, w/v%) and then homogenized to yield MA-BC dispersions at 0.4 w/v%; among these, the 1:30 MA-BC dispersion showed the most favorable balance of carboxyl content (0.15 mmol/g), smallest nanofiber diameter which was found as 26.7 nm, highest crystallinity (93%), and stable, moderately hydrophilic wettability of 71°. When this 1:30 MA-BC dispersion was combined with 10 w/v% gelatin and EDC/NHS crosslinked, the resulting MA-BC–GEL gels displayed shear-thinning behavior, low tan δ of 0.2, and a compression modulus of 0.72 MPa indicating a printable and mechanically competent bio-ink. Compared with neat BC, 1:30 MA-BC significantly enhanced MC3T3-E1 cell viability. On day 7 of cell culture studies, 1:30 MA-BC group upregulated osteogenic markers (ALP, RUNX2, COL1) and yielded more mineralized nodules, demonstrating a strong pro-osteogenic effect. Overall, MA treatment turned dense, poorly printable BC into well-dispersed, nanofibrillar MA-BC that, at an optimized 1:30 ratio, provided rheological control, mechanical support, and osteo-inductive signaling needed to transform a simple gelatin gel into a robust, bone-oriented bio-ink for 3D printing (Wang et al., 2024).

Iglesias-Mejuto et al. (2025) combined 3D printing and X-aerogel technology to obtain polyurea-cross-linked 3D printed cellulose aerogels for tissue engineering. 12 v/v% of MC inks were printed either alone or with 6 mg/mL of BC nanofibers to give MC and BC/MC hydrogels, which were then converted into alcogels and minimally cross-linked with an aliphatic triisocyanate (Desmodur N3300) to form an ultra-thin polyurea shell on the cellulose skeleton. The cellulose network of MC and BC nanofibers provided the primary porous backbone. All aerogels displayed very high porosity of 90%, hierarchical meso–macro porosity (BET ≈257–275 m^2^/g, mesopores of 12–21 nm), and high print fidelity, with BC nanofibers further stiffened the skeleton and reducing shrinkage relative to MC alone. Polyurea nano-cross-linking, despite being present in barely detectable amounts, rigidized the cellulose framework, lowering volume shrinkage, improving skeletal density, and dramatically enhancing dimensional stability in water and physiological conditions. The cross-linked aerogels absorbed 1,500%–1800% water by weight yet remained intact for at least 10 days. On day 3, cell studies showed high NIH/3T3 viability, no hemolysis, no irritation in the HET-CAM assay, and no acute toxicity in the *Artemia salina* model, confirming that the 3D printed MC/BC nanofiber aerogels, minimally reinforced with polyurea, are structurally robust, highly porous, and cytocompatible scaffolds with strong potential for tissue engineering (Iglesias-Mejuto et al., 2025).

Turlybekuly et al. (2020) reported an inkjet-printing approach to fabricate composite scaffolds based on BC and HAp for bone engineering. Printable inks were prepared by dispersing BC nanoparticles in aqueous CaCl_2_/Na_2_HPO_4_ solutions with different proportions, so that HAp precipitated *in situ* within the BC network during or after printing. The dried structures were then obtained by freeze-drying, yielding porous printed constructs. BC acted as the continuous nanofibrous matrix, providing the 3D network in which HAp particles nucleated and distributed. Microstructural analysis showed a homogeneous dispersion of HAp within the BC matrix, rather than phase separation. In this system, BC is essentially the structural “backbone” that confers shape, printability and a hydrated, ECM-like environment, while HAp supplies the mineral phase required for osteoconductivity. The work overall demonstrated that BC is a suitable printable carrier for HAp, enabling inkjet-printed BC/HAp scaffolds with uniform mineral distribution that are promising for bone defect filling.

Sabio et al. (2023) demonstrated a living-material “probiotic cellulose” (PC) by embedding probiotics within a BC scaffold, then allowing bacterial proliferation *in situ* to gradually change the material’s viscoelastic properties. The BC matrix serves as a hydrated, nanofibrillar backbone that supports bacterial viability. Over time and with increasing probiotic density, the material transitions from a relatively fluid, low-viscosity behavior to a more solid-like, elastic response, effectively “tuning” viscoelasticity via living activity rather than external stimuli. This dynamic modulation offers a bio-ink paradigm where mechanical properties evolve with time, potentially allowing self-adapting, self-healing or responsive scaffolds. The study highlights how combining cellulose’s structural properties with living organisms can create a new class of functional biomaterials with built-in adaptability, a promising direction for future 3D printed living scaffolds.

### 2.3 3D printed CNFs for bone tissue engineering applications

TEMPO-oxidized cellulose nanofibers (TOCNFs) and native CNF play markedly different roles in scaffold design due to their distinct chemistries. TOCNFs possess abundant surface carboxylate groups, giving them strong ionic affinity toward Ca^2+^, pronounced shear-responsive rheology, and superior stabilization of printed architectures. In contrast, native CNFs and microfibrous cellulose mainly contribute through mechanical reinforcement and structural templating, rather than mineralization (Isogai et al., 2011). In systems employing TOCNFs, the oxidized nanofibers act as the principal rheological modifier and structural backbone of the bioink. Across these systems, TOCNFs consistently enhance the mechanical stability of the printed scaffolds by forming an interconnected nanofibrillar skeleton that bears load under compression or tension (Håkansson et al., 2016). Table 3 shows CNF-based scaffolds for bone tissue engineering.

**TABLE 3:** 3D printed CNF incorporated scaffolds for bone tissue engineering applications.

Main component	Composition (printing method, temperature)	Benefits of CNF
HAp	TEMPO oxidized CNF/HAp (DIW, 25 °C)	supports mineralization (Korkeamäki et al., 2024)
	70 wt% HAp microfibrous cellulose (EHDP, ambient temperature)	ALP activity is higher than non-fibrous scaffolds (Kim et al., 2018)
Alginate	TEMPO oxidized CNF/alginate (DIW, 25 °C)	CNF improved printability, mechanical stability and bioactivity. CNF entangled network enhanced rheology and shape fidelity (Abouzeid et al., 2018)
	TOCNF/alginate (DIW, 25 °C)	TOCNF enhanced rheological properties, viscoelasticity, filament stability and enhanced tensile strength h (Taha et al., 2024)
	3 wt% CNF/alginate/polyacrylamide (DIW, 25 °C)	3% CNF improved tensile strength, toughness, improved shape fidelity, maintain ECM-like microenvironment (Goyal et al., 2025)
Fmoc	Fmoc with CNF (DIW, 25 °C)	CNF led to mechanical reinforcement and rheological properties (Netti et al., 2025)
Photopolymer resin	1, 2 wt% CNF/photopolymer resin (SLA, ambient)	1 wt% CNF doubled tensile strength and increased stiffness (Vidakis et al., 2022)

In alginate/TOCNF hydrogels, exposure to SBF results in the formation of continuous HAp-like mineral layers that reflect the strong osteoconductive capacity of TOCNF-containing formulations (Abouzeid et al., 2018). In alginate/TOCNF filaments, mineralization begins during CaCl_2_ crosslinking, where TOCNFs catalyze the formation of Ca-phosphate phases, later confirmed by SEM, XRD, and EDX (Taha et al., 2024). TOCNF-based aerogels likewise support controlled mineral distribution, yielding scaffolds with enhanced osteoconductivity and tunable bioactivity. These mineralization behaviors cannot be replicated using native CNFs, confirming the central role of TOCNF chemistry in nucleation-driven osteogenesis (Korkeamäki et al., 2024).

Im et al. (2022) developed an osteogenic 3D-bioprintable bioink composed of 1.5% alginate, 1.5% TOCNF, and 0.5% polydopamine nanoparticles to create a structurally stable and biologically active scaffold for bone tissue engineering. TOCNF provided strong shear-thinning behavior and high viscosity recovery, enabling excellent filament formation and preventing structural collapse during printing. After crosslinking, CNF-reinforced scaffolds maintained robust 3D architecture with improved mechanical stability compared to alginate alone. When combined with polydopamine nanoparticles, the cellulose-containing scaffolds significantly enhanced osteogenic differentiation of encapsulated osteoblasts, increasing ALP activity, mineral deposition, and expression of bone-related genes RUNX2, OPN, OCN on day 7. Alizarin red staining for calcified bone matrix was also enhanced on day 14. Overall, the study demonstrated that CNFs act as the key structural and functional component, transforming alginate into a printable, mechanically stable, and osteoinductive bioink suitable for bone tissue engineering.

Native CNFs, in contrast, influence scaffolds primarily through mechanical and architectural mechanisms rather than ionic interactions. Because these CNFs lack the high COO^−^ density characteristic of TOCNFs, they contribute chiefly by creating a flexible but robust fibrillar network that dissipates mechanical energy and maintains printed geometry (Netti et al., 2025). In this context, native CNFs function as structural co-assembling agents that support biological performance through physical reinforcement rather than mineralization. Goyal et al. (2025) designed 3D printed double-network hydrogels based on a polyacrylamide/alginate matrix reinforced with CNF and crosslinked by UV (PAM) plus Fe^3+^ ions (alginate/COO^−^), to obtain tough, printable scaffolds for tissue engineering. Systematically varying CNF content, they showed that around 3 wt% CNF (PAM/ALG1.5/3CNF) is optimal, markedly enhancing tensile strength (∼285 kPa) and toughness (∼200 kJ/m^3^) while maintaining high swelling, thermal stability, and structural integrity after printing. CNFs act as a reinforcing, anisotropy-inducing phase. They form a percolated fibrillar network within the hydrogel, improve viscoelastic behavior and shape fidelity during extrusion, and allow tuning of direction-dependent mechanical properties via CNF orientation. *In vitro* assays confirmed excellent cell compatibility, with CNF-reinforced double network hydrogels supporting high cell viability and proliferation, indicating that the CNF phase not only strengthens the network but also helps create a cytocompatible, ECM-like environment. Overall, this work shows that moderate CNF loading in an iron-crosslinked double network hydrogel system is a powerful strategy to couple printability, toughness, and cellular viability in hydrogel scaffolds.

A distinct case is presented by scaffolds fabricated using electric-field–assisted electrohydrodynamic printing, where microfibrous cellulose is combined with calcium-deficient HAp to form highly ceramic-rich composite architectures. Electric-field–assisted electrohydrodynamic printing (EHDP) was also utilized to fabricate 3D printed CNF-based scaffolds. Kim et al. (Kim et al., 2018) fabricated cellulose/calcium-deficient HAp composite scaffolds using EHDP technique. In this process, cellulose served as the organic carrier and fibrous template, enabling the stable deposition of a high ceramic loading of 70 wt% HAp into a controlled porous structure. The applied electric field produced HAp/microfibrous cellulose bundles arranged into a 3D mesh, whereas conventional extrusion (without ethanol bath and electric field) resulted in non-fibrous, smooth struts despite similar pore sizes. Although protein adsorption and proliferation of MC3T3-E1 pre-osteoblasts were comparable between the two scaffold types, the fibrous cellulose/CDHA scaffolds elicited markedly higher ALP activity on day 7. On day 14, calcium deposition, and Ca/P ratios indicated enhanced osteogenic differentiation. Overall, the study demonstrated that electric-field-induced cellulose microfibers provided a bone-mimetic architecture that significantly improved the osteoinductive performance of CDHA-based scaffolds.

Netti et al. (2025) developed a fully free bioink by combining self-assembling peptides of 9-fluorenylmethoxycarbonyl (Fmoc)- diphenylalanine (FF) with 5 mg/mL CNFs, which self-assemble into a stable, non-crosslinked fibrillar network. The CNF network provides mechanical reinforcement and rheological properties that allow extrusion-based 3D printing without additional chemical crosslinkers. This composite hydrogel forms an ECM-like nanofibrillar matrix with structural integrity and self-supporting capacity. When human osteoblast-like cells were incorporated, the printed scaffolds supported over 95% cell viability, confirming high cytocompatibility. Therefore, the CNF phase is the vital component that transforms a self-assembling peptide hydrogel into a mechanically stable, printable, and cytocompatible bioink suitable for advanced tissue engineering.

**FIGURE 6:**
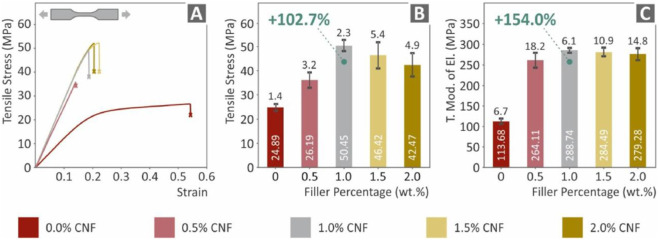
Tensile results **(A)** Typical stress (MPa) vs. strain (mm/mm) curves of Biomed Clear and its CNF nanocomposites, **(B)** Average tensile strength (MPa) and deviations vs. CNF concentration, **(C)** Average tensile modulus of elasticity and deviations vs. CNF concentration (Vidakis et al., 2022). Reproduced from Ref (Vidakis et al., 2022). under the terms of the Creative Commons CC-BY license.

Vidakis et al. (2022) blended a commercial medical-grade photopolymer resin (DSM Somos® BioClear) with upto 2 wt% CNFs to create a nanocomposite suitable for SLA 3D printing. As shown in Figure 6, upon printing, 1 wt% CNF reinforced resin exhibited more than double the tensile strength and significantly increased stiffness compared to the neat resin. The nanofibrillar cellulose acted as an effective reinforcing phase, forming a well-dispersed network that improved mechanical and thermomechanical integrity. While the increased rigidity reduced toughness, the composite maintained structural fidelity and thermal stability under processing conditions. Overall, the CNF phase is the key additive that transforms a standard medical-grade resin into a high-performance, mechanically reinforced 3D printable material, broadening the applicability of photopolymer-based biomedical scaffolds.

Taken together, the distinction between TEMPO-oxidized and native CNF is fundamental to understanding their behavior in tissue engineering scaffolds. TOCNFs dominate in formulations where rheology, Ca-phosphate nucleation, and osteogenic signaling are critical, while native CNFs and microfibrous cellulose excel in systems where mechanical robustness, anisotropic architecture, or compatibility with unconventional matrices such as peptide hydrogels or photopolymers are required. Both classes contribute uniquely to the design of bone-mimetic scaffolds but through mechanisms defined directly by their chemistry and morphology.

### 2.4 Soluble, chemically modified cellulose (CMC, MC and DAEA) as 3D printing enabling components

Across diverse formulations, studies consistently demonstrate that soluble, chemically modified cellulose types including CMC, MC and diethylaminoethyl-modified cellulose (DEAE) act as the central enabling components in 3D printed bone tissue engineering scaffolds. Although their specific functions vary with composition, a unifying trend emerges. CMC and MC transform otherwise unprintable, mechanically unstable, or biologically inert systems into dimensionally stable, highly hydrated, and cytocompatible scaffolds (Abbasi-Ravasjani et al., 2022). Table 4 shows list of such scaffolds for bone tissue engineering applications.

**TABLE 4:** 3D printed soluble, chemically modified cellulose-derived incorporated scaffolds for bone tissue engineering applications.

Main additional component	Composition	Benefits of CMC and MC
PCL	PCL functionalized with sulfated CMC/PCL (DIW, 37 °C)	Increased surface roughness, hardness, hydrophilicity, improved MC3T3-E1 cell adhesion, ALP activity (Abbasi-Ravasjani et al., 2022)
Gelatin	7.5 wt% CMC/HAp/gelatin (DIW, 25 °C–37 °C)	CMC as viscosity enhancer and for structural integrity (Arıcı et al., 2025b)
Alginate	CMC/Bioglass/alginate (DIW, 20 °C–25 °C)	CMC acts as rheology modifier and for shear thinning (Tahmas et al., 2023)
	CMC/Alginate (DIW, 20 °C–25 °C)	Improved viscosity, shear thinning, stabilizes the ink (Abbasi-Ravasjani et al., 2022)
	CMC/CaP/alginate (DIW, 20 °C–25 °C)	CMC act as printable, flexible binder (Wattanaanek et al., 2022)
	MC/Alginate/gelatin/HAp (DIW, 25 °C)	MC increased viscosity, shear thinning, improved flexibility, MC supported cell attachment (Karaca et al., 2025)
Chitosan	BG coated 1 % CMC glycol chitosan (DIW, 20 °C–25 °C)	CMC enable self gelation and print fidelity (Janarthanan et al., 2020)
	MC/trimethyl chitosan silicate BG (DIW, 25 °C–37 °C)	Increased viscosity, shear thinning, shape fidelity (Fermani et al., 2021)
PEEK	CMC/PEEK (FDM, 340 °C–400 °C)	CMC provide shear thinning and viscosity (Mughal et al., 2024)
NCF	1.5 NCF/6 wt% CMC (DIW, 20 °C–25 °C)	Dual network, ECM mimetic platform (Mohan et al., 2020)

**FIGURE 7:**
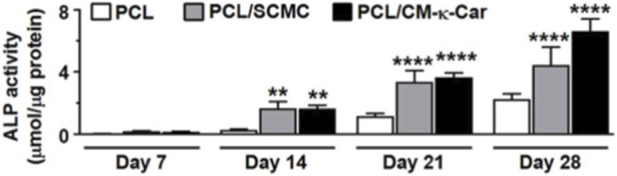
Quantification of ALP activity. Values are mean ± SD (*n* = 3). **Significantly different from control 3D printed PCL scaffolds, *p* < 0.005, ****p < 0.0001. PC; SCMC, sulfated CMC; CM-к-Car, carboxymethyl κ-carrageenan (Abbasi-Ravasjani et al., 2022). Reproduced from (Abbasi-Ravasjani et al., 2022) under the Creative Commons CC-BY license.

Abbasi-Ravasjani et al. (Abbasi-Ravasjani et al., 2022) functionalized 3D printed PCL scaffolds with sulfated CMC or carboxymethyl κ-carrageenan (CM-к-Car) to overcome the poor bioactivity of neat PCL. Surface immobilization of sulfated CMC did not alter scaffold geometry but increased surface roughness, hardness, negative charge, and hydrophilicity, while slightly reducing the bulk elastic modulus. The coating enhanced protein adsorption and significantly improved MC3T3-E1 pre-osteoblast adhesion, proliferation, collagen production, ALP activity, and matrix mineralization compared with unmodified PCL. Figure 7 quantifies ALP activity of the scaffolds up to day 28. Sulfated CMC-functionalized scaffolds most strongly promoted cell proliferation and collagen deposition. Overall, the work showed that sulfated CMC, when used as a surface coating, is a powerful modulator of scaffold hydrophilicity and proliferation, and that fine-tuning polysaccharide chemistry allows differential control over pre-osteoblast growth versus osteogenic maturation on 3D printed PCL.

Arıcı et al. (Arıcı et al., 2025a) showed that CMC was the key component enabling 3D printing of HA/Gel hydrogels by providing the necessary viscosity, shear-thinning behavior, and structural stability for extrusion. CMC formed hydrogen-bond and ionic interactions with gelatin and HAp, creating a cohesive network that improved filament integrity, prevented collapse, and maintained shape fidelity across all compositions. While higher Gel and HAp contents increased viscosity and printing pressures, CMC ensured uniform, stable strut formation and allowed the 7.5% Gel formulations to be printed at ∼40 °C. Overall, CMC acted as the rheological backbone that transformed HAp/Gel mixtures into printable, stable hydrogel inks for bone scaffold fabrication. In their follow up study, 3D printed nano-HA/Gel/CMC bone scaffolds using gelatin (7.5 or 15%), 10 or 20 wt% HAp, and a constant 7.5% CMC as part of an extrusion bioink, then EDC/NHS-crosslinked the constructs (Arıcı et al., 2025b). In this formulation, CMC primarily acted as a viscosity enhancer and rheology modifier. It increased ink viscosity and shear-thinning behavior, which improved filament formation, printability, and shape fidelity of the HAp/Gel constructs, allowing stable printing. The CMC phase also contributed hydrophilicity and water uptake, providing a hydrated environment that supported MC3T3-E1 pre-osteoblast adhesion and proliferation across all compositions. Mechanically, the main strengthening effect came from the combination of 10 wt% HAp and higher Gel content (15%), but CMC was essential to maintain structural integrity and prevent collapse or excessive swelling in these more heavily mineralized formulations. HA10/G15/C7.5 group reached a compressive strength of ∼2.6 MPa, within the trabecular bone range. FTIR, XRD, SBF immersion and alizarin red staining showed that HAp containing Gel/CMC scaffolds developed abundant apatite, and HAp supported by the CMC-rich hydrogel matrix. In summary, CMC did the “enabling” work in this system. It turned HAp/Gel into a printable, dimensionally stable, hydrophilic scaffold that can carry high mineral loadings while supporting good mechanics and robust osteogenic mineralization.

Tahmas et al. (2023) incorporated 58S bioglass particles into an alginate/CMC bioink to produce 3D-bioprinted scaffolds for bone tissue engineering. CMC served as the critical rheology modifier, increasing viscosity, shear-thinning behavior, and structural cohesion of the alginate ink properties essential for stable extrusion and high-fidelity printing. The presence of CMC improved filament definition, reduced spreading, and maintained the mechanical integrity of printed strands, even when 58S bioglass increased the solid content of the ink. 58S bioglass enhanced bioactivity and mineralization, while the CMC–alginate matrix provided a hydrophilic and cell-compatible environment that supported osteoblast adhesion and proliferation. Overall, CMC was again the enabling component that transformed alginate/bioglass mixtures into printable, structurally stable, and biologically supportive scaffolds for bone regeneration.

Wattanaanek et al. (2022) fabricated a 3D printed composite scaffold by combining an alginate/CMC hydrogel matrix with 13–23 wt% amorphous CaP and calcium sulfate hemihydrate fillers, producing multilayer, porous constructs via direct ink writing. The alginate/CMC phase served as a printable, flexible binder network that enabled incorporation of ceramic particles while maintaining porosity of 300–380 µm and structural cohesion. At around 20% inorganic loading, the scaffold exhibited highest compressive strength and compressive modulus while still supporting print fidelity and pore architecture. *In vitro* with MC3T3-E1 osteoblast-like cells, the 18%–20% ACP/CSH composites showed enhanced cell proliferation, indicating cytocompatibility and osteoconductive potential. Thus, the alginate/CMC matrix is crucial in enabling a ceramic-polymer hybrid scaffold that balanced printability, mechanics, porosity, and biological performance.

Janarthanan et al. (2020) formulated a 3D printable, injectable hydrogel by combining 2 w/v% oxidized CMC with glycol chitosan, with the C50G50 composition giving optimal printability and post-printing stability through Schiff-base crosslinking. The CMC-based network produced an interconnected structure with 75% porosity which is suitable for nutrient transport and cell infiltration. Lactoferrin-loaded scaffolds showed sustained protein release for ∼21 days, demonstrating the matrix’s-controlled delivery capability. MC3T3 osteoblasts and BM-MSCs maintained 80%–90% viability, highlighting the cytocompatibility of the CMC/GC system. Overall, CMC phase is the key element that enables self-gelation, print fidelity, porosity, and long-term therapeutic protein release.

Mughal et al. (2024) developed direct ink writing-printed scaffolds by formulating a printable composite ink of PEEK blended with CMC. CMC component provided the critical shear-thinning rheology and viscosity required to enable room-temperature extrusion of PEEK, which is normally only processable at high melt temperatures. After printing, the scaffolds were dip-coated with Zn–Mn–doped mesoporous bioactive glass nanoparticles, imparting strong antibacterial activity and enhanced angiogenic potential. The resulting structures showed a Young’s modulus of ∼2.05 GPa and ∼600 µm pore size with ∼85% porosity, suitable for bone-regeneration applications. Overall, the CMC phase is the key enabler that transforms PEEK into a printable, porous, and biofunctional scaffold for bone tissue engineering.

Mohan et al. (2020) developed a generic route to fabricate self-standing, dual-porous 3D bioscaffolds from aqueous inks composed solely of 1.5 wt% NFC and 6 wt% CMC. NFC provided a nanofibrillar backbone, while CMC strongly interacting with NFC via hydrogen bonding acted as a soluble matrix that tuned viscosity and gel strength. The blend yielded an ink (NC3) with the highest viscosity, storage modulus, and shear-thinning behavior, enabling stable direct-ink-writing with excellent shape fidelity. Direct ink writing followed by freeze-drying produced scaffolds with hierarchical porosity with tunable macropore size of 200–500 µm defined by the print pattern, and interconnected micropores (≈20–90 µm) generated by ice templating. Subsequent dehydrothermal treatment physically cross-linked the cellulose phases, dramatically increasing surface hardness from 0.02 to 0.5 MPa, compressive stress and stiffness up to ∼60 kPa and ∼180 kPa, respectively, and long-term dimensional stability in biofluid, while preserving the cellulosic crystalline structure. hFOB assays showed high viability, widespread cell attachment on the scaffold surface and interior, and even ∼40% higher cell metabolic activity for cross-linked NFC/CMC scaffolds compared with controls, confirming that the cellulose-based dual network provides a cytocompatible, ECM-mimetic, and mechanically tunable platform for bone-related tissue engineering.

In a few studies, MC was also studied for preparation of bone tissue scaffolds by 3D printing (Karaca et al., 2025). Karaca et al. (Karaca et al., 2025) developed bioactive 3D printed MC/Al/Gel/HA scaffolds and showed that MC was the key component enabling printability and structural fidelity. MC significantly increased viscosity and shear-thinning behavior, allowing smooth extrusion, reduced filament spreading, and stable layer formation. It also improved flexibility and inter-strand cohesion, preventing brittleness in HAp-containing formulations and contributing to higher mechanical strength. By creating a hydrophilic, uniform polymer–ceramic network, MC supported improved cell attachment and enhanced the bioactivity of the Gel/HA matrix. Overall, MC served as the primary rheology and structural enhancer, transforming the Al/Gel/HA blend into a printable, flexible, and mechanically reinforced scaffold suitable for bone tissue engineering.

In another study, 3D printed composite scaffolds were produced by using alginate, MC, trimethyl chitosan, and silicate bioactive glass particles to enhance printability and bone-regenerative performance. MC played the key rheological role in the formulation by increasing viscosity and shear-thinning behavior, enabling smooth extrusion and preventing filament spreading, which improved strand definition and overall shape fidelity. MC also contributed to the structural integrity of the printed constructs by forming a more cohesive hydrogel network with alginate and chitosan, stabilizing the incorporation of silicate glass particles. The resulting scaffolds exhibited improved mechanical robustness, uniform pore architecture, and enhanced hydrophilicity. These physical properties supported osteoblast adhesion, viability, and early osteogenic activity. Overall, MC acted as the primary printability and structural enhancer, allowing the multi-component alginate/chitosan/glass system to be fabricated as stable, bioactive bone scaffolds (Fermani et al., 2021).

There is only a single study for use of diethylaminoethyl-modified (DEAE) cellulose for 3D printing tissue engineering scaffolds. Zhang et al. (Vel et al., 2022) developed a 3D printable composite hydrogel based on DEAE-modified cellulose blended with alginate to optimize rheology, mechanical behavior, and cytocompatibility for tissue-engineering applications. DAEA-cellulose was incorporated to provide a positively charged, water-soluble cellulose backbone that strongly enhanced shear-thinning behavior, print fidelity, and filament stability during extrusion. The cationic DEAE groups enabled ionic interactions with alginate’s carboxylate groups, producing a denser, more elastic network after Ca^2+^ crosslinking and significantly increasing compressive modulus and structural integrity relative to alginate alone. Increasing DEAE-cellulose content also improved water retention and resistance to collapse, while maintaining high porosity favorable for nutrient transport. Biological tests demonstrated that the DEAE-cellulose composite hydrogels supported excellent cell viability and spreading, with the moderate DEAE-cellulose formulation showing the best balance between printability, mechanical strength, and cytocompatibility. Overall, DEAE-cellulose acted as the key functional additive regulating rheology, reinforcing the network, improving charge-mediated cell interactions, and enabling the hydrogel to perform reliably as a 3D printable scaffold material.

### 2.5 Cellulose acetate

Owing to its thermoplastic behavior, high viscosity, and favorable cell-interactive surface chemistry, cellulose acetate (CA) is gaining attention as a stand-alone, printability-enabling biomaterial for extrusion-based additive manufacturing. Innovations in hardware and solvent-evaporation–driven stabilization now allow CA to form precise, self-supporting architectures suitable for soft- and hard-tissue scaffold design. Daskalakis et al. (Daskalakis et al., 2025) designed a low-cost, piston-driven extrusion head that enabled 3D bioprinting 20–30 wt% of high-viscosity CA bioinks in acetone at room temperature. In this system, CA itself is the functional biomaterial and rheological driver. At 28 wt%, it provided strong shear-thinning behavior and very high viscosity, which allowed precise line formation, 300 μm pores, and FDM-like resolution without any curing or post-processing. The rapid evaporation of acetone during printing caused CA to form a thin “skin” at the filament surface, stabilizing the extrudate and preserving scaffold geometry, while only minimal shrinkage of 10%–13% was observed. CA’s mechanical robustness and solvent-induced solidification yielded self-supporting, high-resolution scaffolds that supported MSC adhesion, spreading, and migration throughout the construct. Overall, CA acted as a structural, mechanically strong, and printable backbone, demonstrating that CA-based high-viscosity hydrogel inks can be reliably 3D printed into bone-relevant scaffolds when the hardware is appropriately adapted.

Huang and Dean (2020) developed porous tissue-engineering scaffolds using CA as the primary printable polymer for fused-filament–type additive manufacturing. CA served as both the structural matrix and the printability-enabling component. Its thermoplastic behavior allowed controlled melting and extrusion, producing well-defined filaments and uniform pore architectures without collapse. By tuning CA concentration and processing temperature, the scaffolds achieved good dimensional stability, interconnected porosity, and mechanical robustness suitable for soft- and hard-tissue applications. The inherent hydrophilicity and surface chemistry of CA contributed to favorable cell–material interactions, supporting adhesion and proliferation of seeded cells. Overall, cellulose acetate functioned as a mechanically stable, cytocompatible, and highly printable polymer, enabling the fabrication of robust porous scaffolds through standard additive-manufacturing platforms.

## 3 Discussion

The main 3D printing techniques used for bone tissue engineering applications by cellulose derivatives include fused deposition modeling (FDM), direct ink writing (DIW), stereolithography (SLA), inkjet printing, and electrohydrodynamic printing (EHDP), each relying on distinct working principles and material systems. FDM is based on thermoplastic melt extrusion and provides high mechanical strength suitable for load-bearing applications (N’Gatta et al., 2022; Leonovich et al., 2023; Alemán‐Domínguez et al., 2019; N’Gatta et al., 2025; Averianov et al., 2022; Giubilini et al., 2023; Janmohammadi et al., 2025; Babi et al., 2021; Ramanathan et al., 2023; Cakmak et al., 2020; Jiang et al., 2025; Wu et al., 2022; Celestino et al., 2023; Mughal et al., 2024), whereas DIW enables the printing of shear-thinning, cell-compatible hydrogels and represents the most widely used approach (Dobaj Štiglic et al., 2023; Mahmoudi et al., 2025; Dutta et al., 2021; Patel et al., 2021; Maturavongsadit et al., 2021; Patel et al., 2022; Wang et al., 2021; Wang et al., 2024; Aki et al., 2020; Iglesias-Mejuto et al., 2025; Sabio et al., 2023; Abouzeid et al., 2018; Taha et al., 2024; Korkeamäki et al., 2024; Netti et al., 2025; Goyal et al., 2025; Abbasi-Ravasjani et al., 2022; Wattanaanek et al., 2022; Janarthanan et al., 2020; Karaca et al., 2025). SLA offers high resolution through photopolymerization but is limited in biomaterial compatibility (Vidakis et al., 2022), while inkjet printing and EHDP allow controlled deposition and micro/nanoscale structuring, respectively (Liu et al., 2024; Kim et al., 2018). A detailed summary is provided in Supplementary Table S2.

**FIGURE 8:**
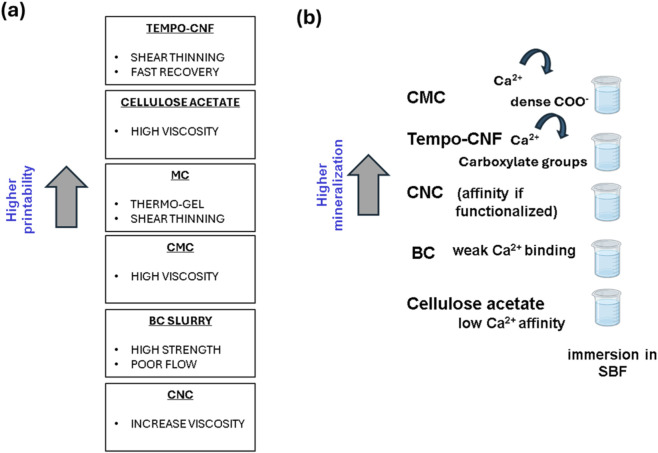
Benefits of different types of cellulose (Sharma et al., 2025) **(a)** printability, **(b)** mineralization potential (Xu et al., 2013; Abouzeid et al., 2018) (drawn by Biorender and Power Point).

The studies reviewed here make it clear that the role of cellulose in 3D printed bone scaffolds is dictated primarily by surface chemistry. Figure 8a,b show benefits of different types of cellulose in terms of printability and mineralization potential, respectively.

As shown in Figure 8a, TEMPO oxidized CNF, CA, and CNC all contribute to printability (Xu et al., 2019). Soluble cellulose derivatives including CMC, MC, and DEAE-cellulose although less crystalline, are crucial rheology modifiers and matrix formers. These soluble cellulose derivatives transform otherwise unprintable systems into shear-thinning, shape-stable inks (Sharma et al., 2025).

As shown in Figure 8b, CMC highly improves mineralization due to its carboxyl groups (Abouzeid et al., 2018). TEMPO-CNF and CNC enhanced mineralization as well (Abouzeid et al., 2018). CNC’s mineralization potential increased if functionalized (Xu et al., 2013). At low loadings, CNC and MCC enhance hydrophilicity and protein adsorption, and promote HAp nucleation, as reported for CNC-reinforced PLA (N’Gatta et al., 2022), CNC- or AgNP-modified PCL (N’Gatta et al., 2025), and MCC/PCL composites (Alemán‐Domínguez et al., 2019). Functionalized CNC variants such as methacrylated CNC (Leonovich et al., 2023), PGA-grafted CNC (Averianov et al., 2022), and acetylated CNC blended with PHBHHx (Giubilini et al., 2023) further improve dispersion within hydrophobic matrices and can participate in covalent or interfacial bonding, leading to enhanced osteogenesis.

TOCNFs, owing to their carboxyl-rich surfaces, additionally act as Ca^2+^ binders and mineralization templates, yielding highly osteoconductive constructs in TOCNF/alginate bioinks (Im et al., 2022; Abouzeid et al., 2018) and TOCNF/HAp aerogels (Korkeamäki et al., 2024). In comparison BC and CA have lower affinity for mineralization, however, their mineralization may be improved by incorporation of other components (Abouzeid et al., 2018).

A more integrative analysis of cellulose type and concentration reveals that optimal performance in 3D printed bone scaffolds is highly dependent on both the physicochemical nature of the cellulose phase and its loading range. For particulate nanocelluloses such as CNC, low concentrations (typically ∼one to three wt%) consistently improve mechanical stiffness, rheological behavior, and bioactivity, as demonstrated in PLA- and PCL-based systems, whereas higher concentrations (≥5 wt%) often lead to agglomeration and reduced mechanical performance due to poor dispersion (N’Gatta et al., 2022; Alemán‐Domínguez et al., 2019; Averianov et al., 2022). Similarly, CNF-based systems exhibit optimal performance at moderate concentrations (e.g., ∼two to five wt%), where an interconnected fibrillar network enhances shear-thinning behavior, mechanical integrity, and print fidelity; however, excessive CNF loading increases viscosity significantly, which can hinder extrusion and lead to processing challenges such as nozzle clogging or poor filament formation (Netti et al., 2025; Goyal et al., 2025). In the case of BC, effective incorporation is typically achieved at lower concentrations (∼0.25–1 wt%), where improvements in hydrophilicity, structural stability, and cell–material interactions are observed (Cakmak et al., 2020; Aki et al., 2020; Celestino et al., 2023). Nevertheless, contradictory mechanical outcomes have been reported, with some studies showing reduced tensile strength upon BC addition. This inconsistency is attributed to factors such as weak interfacial interactions between BC and the polymer matrix and defects in interlayer bonding during printing, indicating that BC performance is strongly dependent on dispersion quality and processing conditions (Cakmak et al., 2020).

In contrast to these reinforcing nanocelluloses, soluble cellulose derivatives such as CMC and MC follow a distinct design paradigm. Across multiple studies, CMC and MC are typically used at higher concentrations (∼5–7.5 wt% or above), where they provide sufficient viscosity, filament stability, and shape fidelity in composite systems such as HAp/Gel, alginate-based, and PEEK-containing scaffolds (Arıcı et al., 2025a; Arıcı et al., 2025b; Tahmas et al., 2023; Mughal et al., 2024; Karaca et al., 2025). However, further increases in their concentration can lead to excessive viscosity, higher extrusion pressures, and reduced printing resolution. Collectively, these observations highlight that optimal cellulose loading is not universal but depends on its functional role, with reinforcing nanocelluloses requiring low-to-moderate concentrations to avoid agglomeration, while soluble derivatives require higher concentrations to achieve printability. Importantly, several limiting cases including nanoparticle agglomeration, excessive viscosity, and interfacial incompatibility further demonstrate that balancing concentration, dispersion, and rheological behavior is critical for achieving optimal scaffold performance.

**FIGURE 9:**
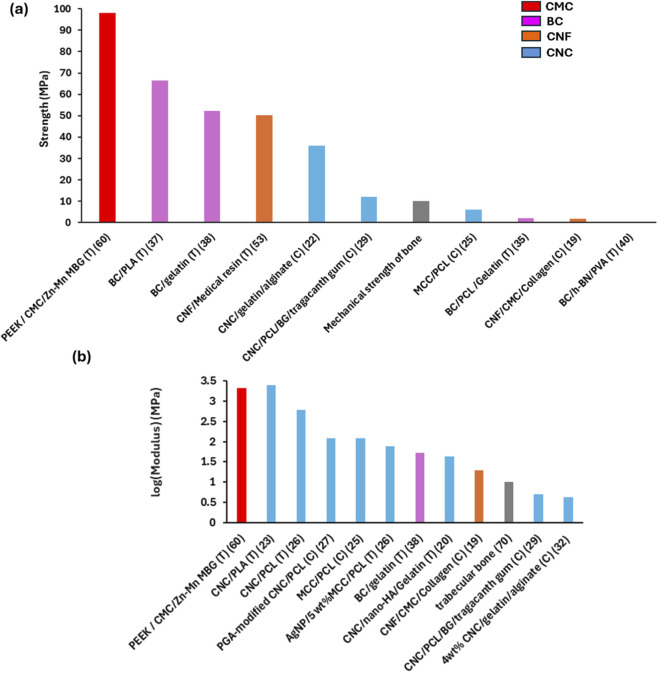
Mechanical properties of the scaffolds **(a)** mechanical strength and **(b)** log (modulus) of the produced scaffolds. (C=Compressive and T = tensile for strength or modulus) (drawn in Microsoft Excel).

The mechanical contribution of cellulose is strongly morphology-dependent. Highly crystalline, particulate forms such as CNC and MCC predominantly enhance stiffness, whereas fibrillar celluloses (BC and CNF) promote toughness through network formation, load transfer, and crack-bridging mechanisms (Omran et al., 2021). In contrast, MC and CMC tend to modify deformability and general mechanical behavior via flexible chain interactions and hydrogen bonding rather than acting as a rigid reinforcement (Antony Jose et al., 2025). Figure 9a,b shows the mechanical strength and log (modulus) of the produced scaffolds, respectively.

According to Figure 9a, BC incorporated scaffolds come forth for enhanced mechanical strength. CNF/medical resin scaffolds also had considerably high strength. CNC incorporated scaffolds have relatively lower mechanical strength within similar polymeric matrices such as PCL. This emphasizes role of BC and CNF for improving mechanical strength. The mechanical strength of non-weight bearing bone is 1–10 MPa (Keaveny et al., 2001). According to this range, most of the scaffolds achieve this range of values. The scaffolds with lower mechanical strength are for cell-laden bioprinting applications such as gelatin or alginate-based scaffolds (Keaveny et al., 2001).

The variation of modulus was high among the research papers; due to this modulus values are presented in log scale and also the modulus values are given in Supplementary Table S1. According to Figure 9b, the modulus was higher for CNC incorporated scaffolds. Additionally, the most important factor for stiffness is the chosen polymer matrix for the 3D printed scaffolds. Consistently, incorporation of CMC in PEEK led to higher mechanical performance which was followed by PLA based scaffolds. PCL also had considerably high performance for CNC and MCC incorporated scaffolds. Human trabecular bone’s stiffness is in between 10 and 3000 MPa (Morgan et al., 2018) and according to this many of the scaffolds fulfill this criterion. On the other hand, the softer platforms are suitable for achieving suitable microenvironments for cell instructive bone regeneration applications (Morgan et al., 2018).

Despite the extensive focus on rheological and mechanical performance, the degradation behavior of cellulose-based scaffolds remains comparatively underexplored, particularly in relation to synchronization with new bone formation. Based on the reviewed studies, degradation characteristics vary significantly depending on cellulose type and structure. Highly crystalline nanocelluloses such as CNC, CNF, and BC exhibit relatively slow degradation under physiological conditions, primarily contributing to long-term structural stability, as observed in PCL/CNC and BC-based composite systems (N’Gatta et al., 2022; Cakmak et al., 2020; Celestino et al., 2023). In contrast, soluble cellulose derivatives such as CMC and MC, due to their amorphous structure and hydrophilic nature, are more prone to swelling and gradual matrix degradation, as demonstrated in HAp/Gel/CMC and alginate/CMC scaffolds, where the cellulose phase supports hydration and remodeling of the scaffold matrix (Arıcı et al., 2025a; Arıcı et al., 2025b; Tahmas et al., 2023; Karaca et al., 2025). CA, as a thermoplastic or solvent-processed cellulose derivative, is primarily reported as a structurally stable matrix forming self-supporting architectures through solvent evaporation–induced solidification, with minimal shrinkage and high dimensional stability (Daskalakis et al., 2025). However, its degradation behavior is less frequently characterized in the context of bone regeneration, and is expected to depend strongly on processing conditions and scaffold architecture rather than intrinsic rapid resorption.

Importantly, degradation behavior is not governed solely by the cellulose phase but by the overall composite system. The incorporation of bioactive components such as hydroxyapatite or bioactive glass can promote mineral deposition and tissue ingrowth, partially compensating for slow polymer degradation and supporting scaffold integration (Janmohammadi et al., 2025; Turlybekuly et al., 2020; Tahmas et al., 2023). However, systematic studies directly correlating cellulose degradation rates and degradation products with *in vivo* bone regeneration and immune response remain limited. Therefore, achieving an optimal balance between structural stability and controlled degradation remains a key design challenge for cellulose-based scaffolds and represents an important direction for future research.

**FIGURE 10:**
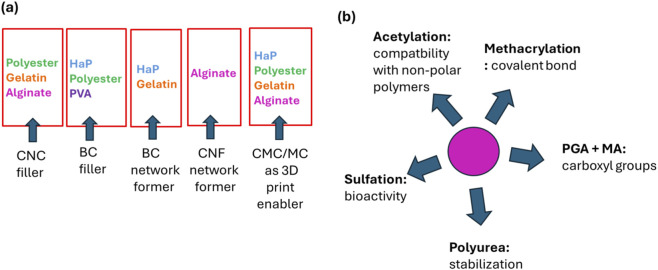
**(a)** Type of cellulose used with common polymers during 3D printing of bone scaffolds **(b)** cellulose functionalization strategies for 3D printing bone scaffolds.

Figure 10 shows (a) the cellulose phases used with various common polymers and (b) its functionalization.

As observed in Figure 10a, additional polymer or bioceramic selection strongly depends on choice of cellulose phase. CNCs integrate best with hydrophobic polyesters due to their rigid particulate nature. CNFs, TOCNFs and BC form percolated fibrillar networks that dominate hydrogel rheology and printed scaffold architecture (Netti et al., 2025; Goyal et al., 2025). CNF and MFC are predominantly used in soft, hydrogel-based bioinks due to their intrinsic water affinity, entangled fibrillar network, and strong rheology-modifying behavior. CA demonstrates that cellulose can also serve as a primary printable thermoplastic or solvent-processable polymer, producing stable 3D architectures with tunable mechanical and osteoconductive properties (Thakur et al., 2013).

Figure 10b shows that chemical modification of cellulose has proven to be a powerful strategy for enhancing the printability, bioactivity, and translational potential of 3D printed bone scaffolds by directly tailoring surface chemistry and interfacial interactions. Carboxylation-based modifications, including TEMPO oxidation of CNFs, maleic acid modification of BC, and poly (glutamic acid) grafting of CNCs, introduce carboxyl-rich surfaces that improve dispersion, induce shear-thinning rheology, and promote Ca^2+^ binding and apatite nucleation, leading to enhanced osteogenic differentiation *in vitro* (Tortorella et al., 2020). Notably, such strategies have also yielded positive *in vivo* bone regeneration, with significantly increased new bone formation and defect bridging reported for PGA-modified CNCs in PCL scaffolds and acetylated CNCs in PHBHHx scaffolds (Giubilini et al., 2023). Methacrylation of CNCs enables their covalent integration into photocrosslinked polymer networks, preventing filler–matrix decoupling and resulting in improved structural stability and cell adhesion (Leonovich et al., 2023). In contrast, acetylation of CNCs primarily enhances compatibility with hydrophobic polyesters, yielding better dispersion, tunable stiffness–ductility balance, and superior tissue integration *in vivo* (Giubilini et al., 2023). Surface sulfation of CMC, applied as a coating rather than a bulk modifier, selectively increases negative charge density and protein adsorption, thereby enhancing pre-osteoblast proliferation, collagen deposition, and alkaline phosphatase activity without altering scaffold geometry (Abbasi-Ravasjani et al., 2022).

Hybrid multi-material printing has emerged as an important strategy in cellulose-based scaffold design. Emerging approaches also include multi-material and gradient architectures, as well as early-stage 4D printing concepts, where chemically modified cellulose systems enable time-dependent or stimuli-responsive behavior (Wang et al., 2024; Sabio et al., 2023). Looking forward, integrating chemically modified cellulose into multi-material, gradient, and 4D-printed scaffolds offers a promising route to dynamically regulate mechanics and bioactivity during bone healing.

In many of the systems discussed in this review, cellulose is integrated with polymers, bioceramics, or bioactive components, where it functions as a rheology modifier, reinforcing phase, or interfacial mediator (N’Gatta et al., 2022; Alemán‐Domínguez et al., 2019; Cakmak et al., 2020; Tahmas et al., 2023). In particular, incorporation of bioactive ions (e.g., Ca^2+^ and related therapeutic ions) through phases such as calcium phosphates or bioactive glasses enables additional control over osteogenic and biological responses (Janmohammadi et al., 2025; Turlybekuly et al., 2020; Tahmas et al., 2023). Nonetheless, in addition to HAp, future studies should also consider alternative calcium phosphate phases such as tricalcium phosphate (TCP), octacalcium phosphate (OCP), and related compounds, which offer tunable degradation behavior and can be incorporated either as pre-synthesized particles or via *in situ* precipitation during scaffold fabrication.

Furthermore, recent studies demonstrate the feasibility of cellulose-based bioinks, especially CNF-containing systems, for cell-laden bioprinting, although such applications remain limited compared to conventional bioinks (Im et al., 2022; Netti et al., 2025). Another important limitation in the current literature is the lack of standardized rheological characterization and reporting protocols for cellulose-based bioinks. Table 5 shows viscosity, G′ and G″ values for rheological studies of the studied biomaterials. To improve comparability, rheological values were selected at similar shear rates (∼1 s^-1^) and frequencies (∼1 rad/s or 1 Hz) where available. As shown in Table 5, most cellulose-based bioinks exhibit viscosities in the range of ∼10^2^–10^3^ Pa s under these conditions, consistent with requirements for extrusion-based 3D printing. In addition, most systems demonstrate dominant elastic behavior (G′ > G″), typically by one order of magnitude, indicating stable gel-like networks capable of maintaining shape fidelity after deposition.

**TABLE 5:** Viscosity, G′ and G″ values for rheological studies of the studied biomaterials.

Biomaterial choice	Viscosity η (Pa.s)	G’ (Pa)	G’’ (Pa)
CCS/SF/CNPs (Patel et al., 2022)	104	2 × 10^4^ at 10 rad/s	7 × 10^3^ at 10 rad/s
TO-BC/GEL (Wang et al., 2021)	5 × 10^2^ at 1s^-1^	3 × 10^3^ at 1 rad/s	3 × 10^2^ at 1 rad/s
MA-BC-GEL (Wang et al., 2024)	10^2^ at 1s-1	10^5^ at 1 rad/s	10^4^ at 1 rad/s
SA-TOCN (Taha et al., 2024)	1 at 100 s^-1^	2.5 × 10^3^ at 1% strain	3 × 10^2^ at 1% strain
Fmoc-FF/CNF (Netti et al., 2025)	10^2^	10^4^ at 1 Hz	10^3^ at 1 Hz
AM/ALG/CNF (Goyal et al. 2025)	0.8 at 100 s^-1^	500 at 10 rad/s	50 at 10 rad/s
HaP/CMC/GEL (Arıcı et al., 2025a)	10^3^ at 1s^-1^	10^4^ at 1 rad/s	10^3^ at 1 rad/s
PEEK/CMC/Bioactive glass (Mughal et al., 2024)	3 × 10^5^ at 1 s^-1^	N/A	N/A
HaP/MC/GEL (Karaca et al., 2025)	100 at 1s-1	10^3^ at 1 rad/s	10^2^ at 1 rad/s
Alg/MC/TCM (Fermani et al., 2021)	500 at 1 s-1	10^4^ at 1 Hz	10^3^ at 1 Hz
Cellulose acetate (Daskalakis et al., 2025)	100 at 1 s-1	N/A	N/A
CNF/GelMA (Xu et al., 2019)	200 at 1 s-1	N/A	N/A

Furthermore, nanofibrillar cellulose systems (e.g., BC and CNF) tend to exhibit higher storage modulus values compared to molecular derivatives such as MC and CMC, suggesting enhanced network reinforcement. However, some variability remains due to differences in experimental conditions and incomplete reporting (e.g., missing G′/G″ data or measurements at non-comparable shear rates), which continues to limit direct cross-study comparison. This reinforces the need for standardized rheological characterization protocols in the field.

Cellulose-based materials offer important advantages from a sustainability perspective, as cellulose is a renewable, abundant, and biodegradable biopolymer. However, the overall environmental impact of cellulose-based scaffold production depends not only on the raw material but also on the processing methods used. Many current approaches involve chemical treatments or solvent-based processing, which may reduce the environmental benefits if not carefully controlled. Therefore, there is increasing interest in developing greener strategies, including aqueous-based systems, reduced solvent usage, and energy-efficient processing routes. In addition, more comprehensive evaluation methods such as life-cycle assessment will be important in future studies to better understand and optimize the sustainability of cellulose-based biomaterials.

Collectively, these studies demonstrate that cellulose modification shifts its role from a passive reinforcement to an active biofunctional component, with carboxylation- and esterification-based strategies showing the strongest evidence for improving both print fidelity and bone regeneration, including validated *in vivo* performance where assessed. However, *in vivo* studies involving cellulose containing systems are scarce. Future work encompassing *in vivo* studies would further clarify the translational potential for cellulose-based scaffolds. From a translational perspective, cellulose-based scaffolds face several important challenges. Biocompatibility evaluation according to standard guidelines (e.g., ISO 10993) is required before clinical use. In particular, plant-derived cellulose nanomaterials such as CNC and CNF may contain residual endotoxins, which can induce undesired inflammatory responses if not properly removed. Therefore, careful purification and testing are necessary, typically involving approaches such as washing, dialysis, or chemical treatments. In addition, sterilization is a critical consideration, as commonly used methods such as autoclaving, gamma irradiation, or ethylene oxide treatment may alter the structural, mechanical, or degradation properties of cellulose-based scaffolds. These effects may include changes in crystallinity, molecular weight, or network integrity depending on the sterilization route. These factors should be carefully controlled to ensure both safety and functional performance during potential clinical translation. Overall, research suggests important role of cellulose in 3D printing of bone tissue engineering scaffolds and its future translational potential.

## 4 Conclusion

This review has shown that cellulose and its derivatives are no longer peripheral “add-ons” but central design tools for 3D printing of bone tissue scaffolds. Across a wide spectrum of systems from PLA and PCL composites to hydrogels, resins, and living materials cellulose phases consistently acted as multifunctional components that improved rheology, mechanics, and biological performance. CNCs generally served as stiff, rod-like reinforcements that strengthened synthetic and natural matrices, increased hydrophilicity and protein adsorption, and promoted HAp nucleation and osteogenic cell responses, even at low loadings. At the network scale, CNFs and BC provided entangled, ECM-like backbones that imparted shear-thinning behavior and structural stability to bioinks, supported high cell viability, and enabled mineralization and, in some cases, immunomodulation or controlled drug delivery.

Chemically modified cellulose derivatives such as CMC and MC expanded the design space further by offering soluble, tunable backbones for crosslinking, injectability, and surface functionalization for 3D printed bone tissue scaffolds. In many formulations, these derivatives were the “enabling” components. They converted otherwise unprintable or unstable inks such as PEEK into shear-thinning, shape-stable systems with adequate porosity and cytocompatibility. Surface-immobilized cellulose-based coatings additionally allowed decoupling of bulk mechanics from bioactivity, providing a means to independently tune pre-osteoblast proliferation versus osteogenic differentiation. The choice of the other polymeric phase also had a high impact on the ultimate mechanical performance and biological response but that is also dictated by the choice of the cellulose phase as the cellulose phase and the other polymeric phase needs to be chemically compatible.

Despite this progress, important challenges remain. Long-term *in vivo* studies directly comparing different cellulose types, loadings, and architectures are still limited, and systematic correlations between cellulose structure, degradation kinetics, immune response, and functional bone regeneration are only beginning to emerge. The field would benefit from standardized reporting of rheological parameters, cellulose characteristics, and *in vitro*/*in vivo* endpoints to allow quantitative comparison across studies. Future work should also explore 4D printing, multi-material gradients, and intelligent, stimuli-responsive cellulose systems that can adapt their mechanical or biochemical cues to different healing phases. Literature suggests that surface functionalization of the cellulose phase would further enhance both the physical and biological responses. If these challenges are addressed, cellulose-based bioinks and 3D printed constructs are well positioned to move from promising laboratory prototypes toward clinically relevant, sustainable solutions for bone repair and regeneration.
