# Supplementary material for: Role of cellulose and its derivatives for 3D printing of bone tissue scaffolds
Source: Front Bioeng Biotechnol. 2026 Jul 6;14:1814617. doi: 10.3389/fbioe.2026.1814617 (PMC13381466; doi:10.3389/fbioe.2026.1814617)
Supplement: Supplementary file 1 [file Supplementaryfile1.docx]

**Supplementary File**

Table S1 shows the stiffness values for the produced scaffolds

**Table S1.** Stiffness values for the produced scaffolds

| **Produced 3D printed scaffolds** | **Stiffness (MPa)** |
| --- | --- |
| PEEK/CMC/Zn-Mn MBG [60] | 2050 |
| CNC/PLA [23] | 2400 |
| PGA-modified CNC/PCL [27] | 122 |
| MCC/PCL [25] | 120 |
| AgNP/5 wt%MCC/PCL [26] | 76 |
| CNC/nano-HA/Gelatin [20] | 43 |
| BC/Gelatin [38] | 52.2 |
| CNF/CMC/Collagen [19] | 20 |
| Trabecular bone [72] | 10-3000 |
| CNC/PCL/BG/tragacanth gum [29] | 5.1 |
| 4wt% CNC/gelatin/alginate [21] | 4.32 |
| CNC/chitosan/silk fibroin [32] | 0.6 |
| CNC/nano-HA [49] | 0.6 |
| BC/gelatin gel [38] | 0.15 |
| CNF/alginate/ polydopamine [31] | 0.00203 |
| Nanocellulose / Chitosan bioink [63] | 0.00012 |

**Table S2.** Printing technique and the exploited biomaterials

| **Printing Technique** | **Working Principle** | **Typical Materials Used in This Review** | **Key Features / Relevance** |
| --- | --- | --- | --- |
| **Fused Deposition Modeling (FDM)** | Melt extrusion of thermoplastic filaments | PCL, PLA, PHB, PHBH + CNC/MCC/BC | High mechanical strength, suitable for load-bearing scaffolds [23 -29], [33-37], [42], [60] |
| **Direct Ink Writing (DIW) (Extrusion-based bioprinting)** | Extrusion of shear-thinning inks at room/moderate temperature | Alginate, gelatin, CMC, MC, CNF, BC, bioactive glass | Most widely used; enables cell-compatible and hydrogel systems [19 -22], [30], [32], [38-41], [44], [47-51], [54], [58-59], [62] |
| **Stereolithography (SLA)** | Photopolymerization of liquid resin using light | Photopolymer resins + CNF | High resolution, limited biomaterial compatibility [53] |
| **Inkjet Printing** | Droplet-based deposition of low-viscosity inks | BC/HAp systems | Good for controlled deposition, lower mechanical strength [45] |
| **Electrohydrodynamic Printing (EHDP)** | Electric field-assisted jetting of fine fibers | Cellulose + calcium-deficient HAp | Enables micro/nano fibrous structures, high resolution [52] |
